# Supplementary material for: Efficient generation of neutral and charged biexcitons in encapsulated WSe2 monolayers
Source: Nat Commun. 2018 Sep 13;9:3718. doi: 10.1038/s41467-018-05917-8 (PMC6137141; doi:10.1038/s41467-018-05917-8)
Supplement: Supplementary file 1 — Supplementary Information [file 41467_2018_5917_MOESM1_ESM.pdf]

Supplementary Information to Efficient generation of neutral and charged biexcitons in encapsulated WSe_2 monolayers

Ziliang Ye,[1,2] Lutz Waldecker,[1] Eric Yue Ma,[1] Daniel Rhodes,[3] Abhinandan Antony,[3] Bumho Kim,[3] Xiao-Xiao Zhang,[1] Minda Deng,[1] Yuxuan Jiang,[4] Zhengguang Lu,[4,5] Dmitry Smirnov,[4] Kenji Watanabe,[6] Takashi Taniguchi,[6] James Hone,[3] and Tony F. Heinz[1,7,*]

[1]*Department of Applied Physics, Stanford University, 348 Via Pueblo Mall, Stanford, California 94305, USA*

[2]*Department of Physics and Astronomy, University of British Columbia, Vancouver, British Columbia V6T 1Z4, Canada*

[3]*Department of Mechanical Engineering, Columbia University, New York, New York 10027, USA*

[4]*National High Magnetic Field Laboratory, Tallahassee, Florida 32310, USA*

[5]*Department of Physics, Florida State University, Tallahassee, Florida 32310, USA*

[6]*National Institute for Materials Science, 1-1 Namiki, Tsukuba 305-0044, Japan*

[7]*SLAC National Accelerator Laboratory, Menlo Park, California 94025, USA*

Supplementary Note 1. Characterizing the radiation pattern of the dark exciton

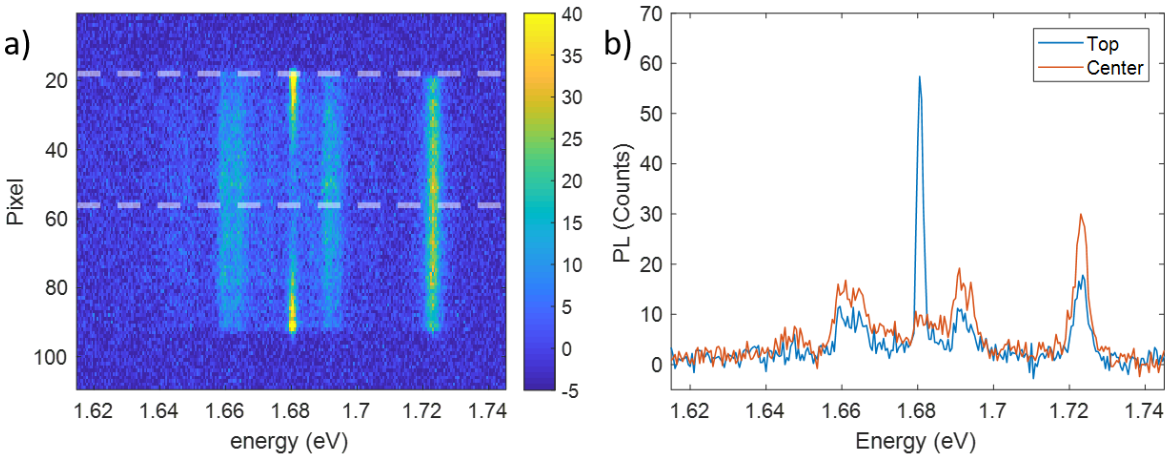

SUPPLEMENTARY FIG. 1. a) A representative spectrograph taken in the back focal plane. The maximal collection angle is 37°. b) A horizontal cross section along the two dashed lines in Fig. 1a, corresponding to PL spectra collected along surface normal at the maximal angles. The out-of-plane dipole of the dark exciton results in higher intensity for increased emission angles relative to the surface normal.

The dark exciton related emission is identified by the radiation pattern of the PL, as imaged in the back focal plane of our collection objective. Using a Bertrand lens, we project the back focal plane onto the slit of an imaging spectrometer and thus measure the radiation pattern of each individual spectral peak [1]. Since the in-plane component of the dark exciton is negligible, it emits through coupling with the out-of-plane dipole of a higher-lying transition [2]. As a result, the dark exciton emits most strongly in the in-plane direction, and has a node in the normal direction, making it easily distinguishable from the emission patterns of any of the bright exciton species (Supplementary Fig. 1).

Supplementary Note 2. Rate equations for calculating the power dependence of the biexciton emission

Here, we consider the expected dependence of the biexciton emission on the exciton density, making use of the experimental intensity dependencies of the neutral and dark

excitons. We start from a system of rate equations that determine the densities of bright and dark exciton, as well as of the biexciton:

$$\frac{\partial \rho_{\mathrm{X}^0}}{\partial t} = -\frac{\rho_{\mathrm{X}^0}}{\tau_{\mathrm{X}^0}} - \gamma \rho_{\mathrm{X}^0}\rho_{\mathrm{X}^\mathrm{D}} + P_{\mathrm{X}^0} \tag{1a}$$

$$\frac{\partial \rho_{\mathrm{X}^\mathrm{D}}}{\partial t} = -\frac{\rho_{\mathrm{X}^\mathrm{D}}}{\tau_{\mathrm{X}^\mathrm{D}}} - \gamma \rho_{\mathrm{X}^0}\rho_{\mathrm{X}^\mathrm{D}} + \frac{\rho_{\mathrm{XX}}}{\tau_{\mathrm{XX}}} + P_{\mathrm{X}^\mathrm{D}} \tag{1b}$$

$$\frac{\partial \rho_{\mathrm{XX}}}{\partial t} = -\frac{\rho_{\mathrm{XX}}}{\tau_{\mathrm{XX}}} + \gamma \rho_{\mathrm{X}^0}\rho_{\mathrm{X}^\mathrm{D}} \tag{1c}$$

where ρ_{X^0}, $\rho_{\mathrm{X}^\mathrm{D}}$ and ρ_{XX} are the densities and τ_{X^0}, $\tau_{\mathrm{X}^\mathrm{D}}$ and τ_{XX} the lifetimes of X^0, X^D and XX and γ is the formation rate of the XX species, which we assume to be a constant. P_{X^0} and $P_{\mathrm{X}^\mathrm{D}}$ are the bright and dark exciton formation rates, which, in the steady-state low density regime, scale with the emission strength and therefore can be written as I^α, where α is 1.22 and 1.03, respectively. We note that in these equations, we have neglected the possible role of the formation of dark biexcitons. In the low density regime, we do not expect this omission to be significant.

Solving for the steady-state density of the excitons, we obtain $\rho_{\mathrm{X}^0} = \frac{P_{\mathrm{X}0}}{\gamma P_{\mathrm{X}^\mathrm{D}}\tau_{\mathrm{X}^\mathrm{D}} + \frac{1}{\tau_{\mathrm{X}0}}}$, $\rho_{\mathrm{X}^\mathrm{D}} = P_{\mathrm{X}^\mathrm{D}}\tau_{\mathrm{X}^\mathrm{D}}$, $\rho_{\mathrm{XX}} = \frac{\gamma \tau_{\mathrm{X}^\mathrm{D}}\tau_{\mathrm{XX}} P_{\mathrm{X}0} P_{\mathrm{X}^\mathrm{D}}}{\gamma P_{\mathrm{X}^\mathrm{D}}\tau_{\mathrm{X}^\mathrm{D}} + \frac{1}{\tau_{\mathrm{X}0}}}$. The biexciton density is proportional to the product $P_{\mathrm{X}^0}P_{\mathrm{X}^\mathrm{D}}$ in the limit where $\frac{\rho_{\mathrm{XX}}}{\rho_{\mathrm{X}0}} \ll \frac{\tau_{\mathrm{XX}}}{\tau_{\mathrm{X}0}}$. In this case, the biexciton emission is expected to scale with intensity as I^α, $\alpha = 2.25$. For higher biexciton densities, its power-dependence will flatten and become linearly proportional to the bright exciton formation rate, P_{X^0}. In our experiment, we are below the saturation regime, as the biexciton density increases nearly quadratically and its emission is weaker than that of the bright exciton.

Supplementary Note 3. Exciton collision and biexciton formation

Here we introduce a simple kinetic model to describe the formation of the observed biexciton. Biexcitons are created when two excitons collide. The formation rate is given accordingly by the collision frequency $\nu = z n_a n_b$,where $n_{a,b}$ are the (2D) densities of the constituent excitons. For thermalized excitons in 2D, we have $z = \sigma_{ab} \cdot \sqrt{(\pi k_B T)/(2\mu_{ab})}$, where σ_{ab} is the collision cross-section, $k_B T$ is the thermal energy, and $\mu_{ab} = m_a m_b/(m_a + m_b) \sim 0.3 m_0$ is the reduced mass of the exciton. As the biexciton is composed of at least one bright exciton, the collision needs to occur within its lifetime τ_{X^0}. Given the radius of

1 nm in the exciton ground state [3], we estimate the collision cross-section as 2 nm. For the laser intensity at which we start to observe the emission from the biexciton feature, the bright exciton emission is 20 times stronger than the biexciton emission, indicating that $n_b/(\nu \cdot \tau_{X^0}) \sim 20$. This suggests the density of the other exciton species n_a is about 370 μm^{-2} (for $\tau_{X^0} = 2$ ps [4]). For our applied laser intensity I $\sim$ 10 Wcm^{-2}, this density level can be achieved for a long-lived dark exciton species.

Supplementary Note 4. Nonequilibrium distribution in the bright exciton pair

Under the strongest applied magnetic fields, the bright exciton pair manifests an inverted population distribution, i.e., the photoluminescence (PL) of the low energy branch (LEB) is weaker than of the low energy branch (HEB). Here, we discuss a mechanism that can lead to such a distribution.

After a bright exciton is initially excited in one valley, one relaxation pathway is through the emission of a K point phonon and scattering of the electron from the upper conduction band to the lower conduction band of the opposite valley with the same spin index. Because the conduction band splitting is comparable to the K point optical phonon energy [5–7], such a relaxation channel is expected to be efficient. On the other hand, if the lower conduction band is already occupied by an electron associated with a long-lived dark exciton, such an intervalley scattering may be suppressed. Since the dark exciton is significantly valley-polarized, as reflected by the emission intensity ratio in the dark exciton pair, the suppression is not symmetric between two valleys. In the steady state, more bright excitons will accumulate in the valley opposite the one with the higher dark exciton population. As a result, the bright excitons can exhibit an inverted intensity distribution, in contrast to the behavior of the dark exciton pair. In addition to this explanation, there are other possible mechanisms for the inverted population distribution of bright excitons that merits further investigation. These include 'hot' K-point phonon [8] activated dark exciton emission and resonant phonon scattering in the presence of the magnetic field.

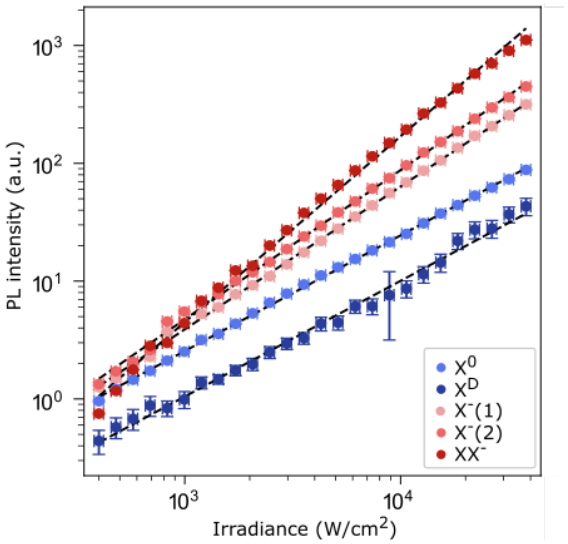

SUPPLEMENTARY FIG. 2. Power dependence of different exciton species in the n-doped regime.

Supplementary Note 5. Power dependence of the exciton species in the n-doped regime

A power-law fit to the PL intensity of the different exciton species in the lightly n-doped regime gives exponents of 0.96 ± 0.01 for X^0, 1.19 ± 0.01 and 1.21 ± 0.01 for the two trion species, respectively, 0.92 ± 0.03 for the dark exciton and 1.56 ± 0.01 for the charged biexciton (Fig. 2). The deviation from the quadratic dependence of XX^- was attributed to the partial equilibrium between the biexciton and the constituent exciton [9]. The slight superlinear power dependence of the trion is expected to have the same origin as that of the exciton in the intrinsic regime, which has been attributed to partial filling of defect states at low laser excitation. As the defects are gradually filled, the loss of excitons/trions to the defect states is reduced, thus giving rise to a superlinear power dependence of the emission intensity.

Supplementary Note 6. Polarization resolved biexciton emission

We also measured the polarization dependence of the biexciton emission (Fig. 3). Clearly the XX and XX^- emission in Fig. 3a and 3b have the same circular polarization as the excitation, confirming their intrinsic nature. In addition, we observe that circular polarization of the photoluminescence is present over the entire spectrum, except for the emission

of the dark exciton, which cannot support circular polarization with its out-of-plane transition dipole moment. We find that the degree of circular polarization is enhanced in the n doped regime and suppressed in the p doped regime (Fig. 3 b & c). No linear polarization is observed in either the XX or XX^{-} peak.

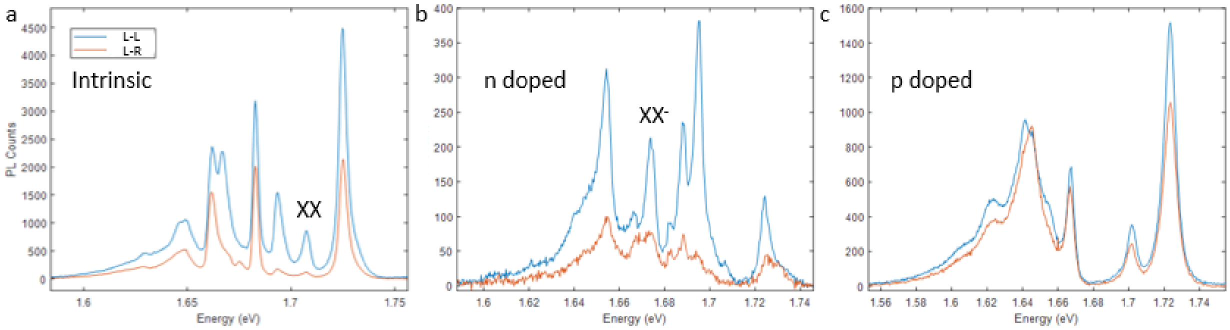

SUPPLEMENTARY FIG. 3. Polarization resolved PL spectrum for different doping regimes. L-L denotes the case where both the excitation and detection are left handed circularly polarized, while L-R stands for the opposite circularly polarized excitation and detection.

* tony.heinz@stanford.edu

[1] Schuller, J. A. *et al.* Orientation of luminescent excitons in layered nanomaterials. *Nature Nanotechnology* **8**, 271–276 (2013).

[2] Wang, G. *et al.* In-Plane Propagation of Light in Transition Metal Dichalcogenide Monolayers: Optical Selection Rules. *Physical Review Letters* **119**, 047401 (2017).

[3] Qiu, D. Y., Felipe, H. & Louie, S. G. Screening and many-body effects in two-dimensional crystals: Monolayer MoS_2. *Physical Review B* **93**, 235435 (2016).

[4] Robert, C. *et al.* Fine structure and lifetime of dark excitons in transition metal dichalcogenide monolayers. *Physical Review B* **96**, 155423 (2017).

[5] Sahin, H. *et al.* Anomalous Raman spectra and thickness-dependent electronic properties of WSe_2. *Physical Review B* **87**, 165409 (2013).

[6] Chang, C. H., Fan, X., Lin, S. H. & Kuo, J. L. Orbital analysis of electronic structure and phonon dispersion in MoS_2, $MoSe_2$, WS_2, and WSe_2 monolayers under strain. *Physical Review B* **88**, 195420 (2013).

[7] Zhang, X.-X., You, Y., Zhao, S. Y. F. & Heinz, T. F. Experimental evidence for dark excitons in monolayer WSe_2. *Physical Review Letters* **115**, 257403 (2015).

[8] Waldecker, L. *et al.* Momentum-resolved view of electron-phonon coupling in multilayer WSe_2. *Physical Review Letters* **119**, 036803 (2017).

[9] You, Y. *et al.* Observation of biexcitons in monolayer WSe_2. *Nature Physics* **11**, 477–482 (2015).
